# Microbiota Transplantation as a Future Novel Therapeutic Strategy Approach

**DOI:** 10.3390/diseases14020042

**Published:** 2026-01-28

**Authors:** Suresh Kumar, Himanshu, Pratibha Gaur, Saheem Ahmad, Paridhi Puri, V. Samuel Raj, Ramendra Pati Pandey

**Affiliations:** 1 National Institute of Biologicals, Ministry of Health and Family Welfare, Government of India, Noida 201309, Uttar Pradesh, India; 2 Graduate Institute of Biomedical Sciences, Chang Gung University, No. 259, Wenhua 1st Road, Guishan District, Taoyuan City 333323, Taiwan; 3 Department of Biotechnology and Microbiology, SRM University, Sonepat 131029, Haryana, India; pratibhagaur@gmail.com (P.G.); directorcd4@srmuniversity.ac.in (V.S.R.); 4 Department of Medical Laboratory Sciences, College of Applied Medical Sciences, University of Hail, Hail 2440, Saudi Arabia; ahmadsaheem@gmail.com; 5 University Centre for Research and Development, Chandigarh University, Mohali 140413, Punjab, India; paridhi.e14948@cumail.in; 6 School of Biosciences and Bioengineering, D Y Patil International University, Pune 411044, Maharashtra, India

**Keywords:** bacterial vaginosis, vaginal microbiota transplantation, fecal microbiota transplantation, treatment approaches, clinical trials development

## Abstract

Bacterial vaginosis (BV) is a leading cause of genital discomfort among women globally, and it arises from dysbiosis of the vaginal ecosystem characterized by the overgrowth of pathogenic bacteria. Current therapeutic strategies primarily rely on antibiotics and/or probiotics, which demonstrate clinical efficacy but are frequently associated with limitations such as antimicrobial resistance, high recurrence rates, and incomplete restoration of a healthy vaginal microbiota. Inspired by the success of fecal microbiota transplantation in gastrointestinal disorders, vaginal microbiome transplantation (VMT) from healthy donors has emerged as a potential alternative therapeutic approach for BV. However, experimental and early clinical studies indicate that VMT efficacy is not uniform across individuals, with considerable inter-individual variability in treatment outcomes. Host genetic factors, baseline vaginal microbial composition, immune status, and environmental influences are likely to modulate therapeutic success, underscoring the need for personalized interventions. This article critically evaluates the shortcomings of existing standardized treatments, highlights the potential advantages and challenges of VMT, and discusses emerging, precision-based therapeutic strategies for BV in light of recent research advances and ongoing clinical trials worldwide.

## 1. Introduction

The vaginal microbiota is a complex community of microorganisms, including bacteria and fungi, that inhabit the vaginal environment, leading to homeostasis. This plays a crucial role in maintaining vaginal health by preventing infections, regulating immune responses, and supporting reproductive health. The composition of vaginal microbiota is influenced by various factors such as hormonal changes, age, sexual activity, and use of medications. Moreover, once the homeostasis becomes disturbed, it creates an imbalance between the opportunistic pathogens [1].

Bacterial vaginosis (BV) is a clinically devastating condition associated with vaginal discharge, itching, burning when urinating, increased vaginal pH, and fishy odor [2]. Nevertheless, researchers estimated that up to 50% of females with BV are asymptomatic [3]. BV manifests as a shift in the vaginal ecosystem from a dominant *Lactobacillus* to obligate and facultative anaerobes, including *Gardnerella*, *Prevotella*, and *Atopobium vaginae* [4]. This is one of the most common microbiological vaginal infections in females of childbearing age of 14–49 years [5,6,7]. Globally, the impact of BV varies considerably from 5 to 70% in females depending upon geography and ethnicity [8]. There is a higher prevalence of this condition in parts of Africa as compared to Europe and Asia [6]. On the other hand, approximately 17.8% to 63.7% of the adult population in India suffers from this condition, while the prevalence in the United States is about 30%. Although BV is not a life-threatening condition, it could impose the risk of many gynecological and obstetric disorders like preterm birth, infertility, vulnerability to sexually transmitted infections, and also predispose a greater risk of infections to the upper genital tract [9].

Antibiotic therapy is the cornerstone of treatment for BV. The most widely prescribed antibiotics for treating BV are clindamycin or metronidazole, which cure only ~70–85% of females within 1 month and are associated with complications as they are suboptimal cures [10,11]. Moreover, there is a significant drawback to these types of treatments, which is high recurrence rates of up to 50% observed within 6 months in most patients [12,13]. Another treatment practice involves the use of probiotics (probiotics are live microorganisms that, when administered in adequate amounts, confer a health benefit on the host). They help maintain or restore microbial balance and support immune and metabolic functions, administered orally or intravaginally to symptomatic patients; however, their effectiveness has shown mixed results. Therefore, researchers suggested using a whole microbiome rather than a single bacterial type to make it more effective to deal with clinical cases [6]. Briefly, microbiome refers to the collective community of microorganisms—including bacteria, viruses, fungi, and archaea—and their genetic material that inhabit a specific environment in the body. These microbial communities play a crucial role in maintaining host physiology, immunity, and overall health. Further, studies have revealed that organs such as the gut and vagina harbor an array of microbes that play an instrumental role in nutrient extraction, metabolism, and immunity [14,15,16].

The global scenario of microbiota replacement as a treatment strategy has also gained significant attention in recent years, with several therapeutic approaches being explored to address a variety of health conditions. One of the most well-established treatments is fecal microbiota transplantation (FMT), which has shown promising therapeutic results and is becoming one of the most successful first-line treatments for treating gastrointestinal conditions by rebalancing the intestinal microbiota in recent years. The success of FMT inspired the researchers to take VMT as an alternative approach by introducing the microbiome from a healthy donor to a recipient on the basis that both gastrointestinal and vaginal tracts encounter a physiologically similar environment and pathogenesis during microbial dysbiosis [7,17]. Dysbiosis is a state of imbalance in the composition, diversity, or function of the normal microbial community within a specific body site. This disruption is often associated with impaired host–microbe interactions and increased susceptibility to disease. In this article, we explored the therapeutic advancements as well as the inherent pitfalls associated with current treatment methods of BV. Furthermore, it delves into the emerging concept of VMT as a promising and innovative strategy for BV management, supported by recent findings from numerous studies. This comprehensive review aims to provide insights into the potential of these novel approaches to improve treatment outcomes and overcome the challenges associated with traditional therapies.

## 2. Fecal Microbiota Transplantation as a Modern Therapeutic Tool

The active gut microbial community is considered an emergent system that interacts naturally with the host and directs the physiological functions of the host, including digestion to immune homeostasis [18,19]. The imbalance of gut ecosystem homeostasis triggers the pathogenesis of serious gastrointestinal diseases. Therefore, the biological therapy of FMT has attracted much interest and is recommended as the most reliable and promising treatment for refractory gastrointestinal diseases [17,20]. FMT is a promising novel therapeutic concept that involves the introduction of a whole microbial community derived from the fecal material of a healthy donor into the gastrointestinal tract of patients using various techniques such as nasogastric tube, upper tract endoscopy, oral capsules, enema, and sigmoidoscopy or colonoscopy to restore gut microbial balance [21,22,23]. A study performed by Zmora et al. revealed that intraduodenal administration of a healthy fecal microbiome caused restoration of healthy microbiota and warded off recurrence, as observed in patients suffering from recurrent *Clostridium difficile* infection (CDI) within 10 weeks of follow-up, as compared to other treatments using the antibiotic vancomycin [24]. Since then, FMT studies have also been performed in other clinical conditions like cardiometabolic disease and inflammatory bowel disease (IBD), and they show the capability to be applied to other diseases in the future [25,26]. FMT has proven high success rates of over 85% in preventing CDI recurrences compared to a tapering regimen (35 to 42 days) of vancomycin, which showed the maximum success rate of around 69% in current antibiotic treatment regimens [27,28,29]. Compared to the standard treatment regime of antibiotics for treating CDI, FMT is generally considered safe, with mild, self-limiting side effects. Furthermore, oral capsules showed superior efficacy to other routes of administration, such as colonoscopy and enema. However, severe CDI patients are less responsive to FMT [23,29]. Therefore, further research is needed to establish the most appropriate and effective clinical protocols for FMT.

Importantly, the therapeutic success of FMT in recurrent *Clostridioides difficile* infection has prompted broader consideration of microbiota transplantation as a potentially universal therapeutic paradigm for dysbiosis-driven diseases. Beyond the gastrointestinal tract, mucosal ecosystems such as the vaginal cavity share fundamental physiological and immunological characteristics with the gut. Both environments are colonized by complex, niche-adapted microbial communities that play a critical role in maintaining epithelial barrier integrity, regulating local immune responses, and preventing pathogen overgrowth through competitive exclusion and metabolite production.

In both the gut and vaginal tract, dysbiosis is characterized by the loss of beneficial commensals, disruption of epithelial barrier function, elevated pH or inflammatory mediators, and increased susceptibility to infection and disease recurrence. Restoration of microbial homeostasis, rather than eradication of individual pathogens, has therefore emerged as a rational therapeutic goal. In this context, microbiota transplantation represents a systems-level intervention capable of re-establishing functional microbial networks, metabolic outputs, and host–microbe immune crosstalk.

These shared principles provide a strong biological rationale for extending the microbiota transplantation concept beyond the gut to the vaginal ecosystem. VMT is thus conceptually grounded in the same framework that underpins FMT: the reconstitution of a stable, protective microbial community to restore mucosal homeostasis and reduce disease recurrence. This theoretical continuity supports the exploration of VMT as a novel therapeutic strategy for recurrent BV and related vaginal dysbiosis-associated conditions.

## 3. Bacterial Vaginosis

In Asian and White females, the vaginal bacteria are dominated by *Lactobacillus* species, including *L. iners* and *L. crispatus*, while *L. iners* tends to dominate the vaginal microbiota of Black and Hispanic females [30]. BV is characterized by a deficiency of lactic acid-producing bacteria and a corresponding increase in anaerobic bacteria, including *Atopobium*, *Gardnerella*, *Megasphera*, *Prevotella*, and *Sneathia* [31,32,33]. Interestingly, many studies have demonstrated a strong link between BV symptoms and a change in vaginal bacterial composition. The most widely accepted methods of diagnosing BV are Amsel’s criteria or Nugent’s score. The Amsel criteria are mainly used as a diagnostic method due to having high specificity in the clinic setting, including pH measurements, inspection of vaginal secretions, visual inspection under microscopy, and the Whiff test, while the Nugent score relies on Gram-stained smear microscopy images of normal flora and is considered more sensitive. To overcome the shortcomings impounded in microscopy and other point-of-care tests (POCTs), DNA sequencing of vaginal fluid has been devised using molecular markers of BV [7,34]. The *Lactobacillus*-dominated vaginal microbiome secretes various antimicrobial substances like lactic acid, *bacteriocins,* and hydrogen peroxide (H_2_O_2_), which protect the host against various potential harmful pathogens [17,35,36,37]. Intriguingly, vaginal fluid contains a lot of glycogen, which is converted by human *alpha-amylase* into simpler carbohydrates that are turned by *Lactobacillus* species into lactic acid and help in maintaining an acidic environment [7,38]. *Lactobacillus* produces bacteriocins, viz., IIa, IIc, LF221A, J46, gassericin T, and type-*A lantibiotic*, which are proteinaceous bacterial substances, known for their anti-bactericidal properties in the host [39,40]. Most vaginal strains of *lactobacilli* release H_2_O_2_, which maintains a healthy vaginal environment. However, their role is continued under investigation in the context of vaginal bacteria protection. Recent studies have shown that microbiota in the cervical vaginal (CV) space regulates cervical epithelial cell function [41,42]. Additionally, a CV space microbiota dominated by *L. crispatus* is correlated with a healthy cervical environment and confers the integrity of the cervical epithelial barrier [42,43]. In addition, *L*. *crispatus*-dominated microbiota is known to increase bacterial Immunoglobulin A (IgA) coating, known to maintain a healthy intestinal microbiome [42,44]. The vaginal fluids in BV exhibit a dramatic loss of lactic acid concentration as well as high levels of acetate, propionate, butyrate, and succinate, increasing vaginal pH by more than 4.5 [43]. Additionally, the breakdown of amino acids into amines such as putrescine and cadaverine contributes to a vaginal fishy odor [45]. The mucosa layer of the vaginal tract becomes thin due to mucosal protein catabolism and secretes a homogeneous discharge [45]. Many studies have also confirmed the marked increase in concentration of chemokines and cytokines like IL-1β, TNFα, IL-6, and IL-8 in the vagina of females suffering from BV [46]. Moreover, a recent study has demonstrated that synthetic bacterial consortia, including microbiota transplantation, reduce vaginal inflammation and regulate the immune response in the Gardnerella vaginosis model. This treatment has resulted in increased levels of anti-inflammatory cytokines and decreased levels of pro-inflammatory cytokines. This could be the game changer in the current scenario where these two unique techniques merge together to provide the treatment against BV [47].

Recent investigations into vaginal microbiome-based therapies have moved beyond preclinical and exploratory case studies toward formal clinical evaluation. Multiple registered trials are currently assessing VMT as a treatment for recurrent or antibiotic-nonresponsive BV. A key multicenter, randomized, placebo-controlled study (ClinicalTrials.gov NCT04517487) is actively recruiting women with recurrent BV to compare donor-derived VMT versus placebo, with estimated completion in late 2026; this trial is designed to evaluate whether VMT can sustainably re-establish a Lactobacillus-dominated vaginal microbiota and improve clinical outcomes in women who have failed conventional therapies. Another ongoing randomized trial is examining the combination of oral metronidazole plus vaginal microbiota transplant versus sterile saline placebo in women with recurrent BV, with a focus on safety and engraftment of beneficial lactobacilli. Together, these studies represent a significant step toward evidence-based evaluation of VMT and related microbiome modulation strategies, reflecting growing interest in microbiota-targeted therapies for difficult-to-treat vaginal dysbiosis [48].

## 4. Current Treatment Regimens for BV

**Figure 1:**
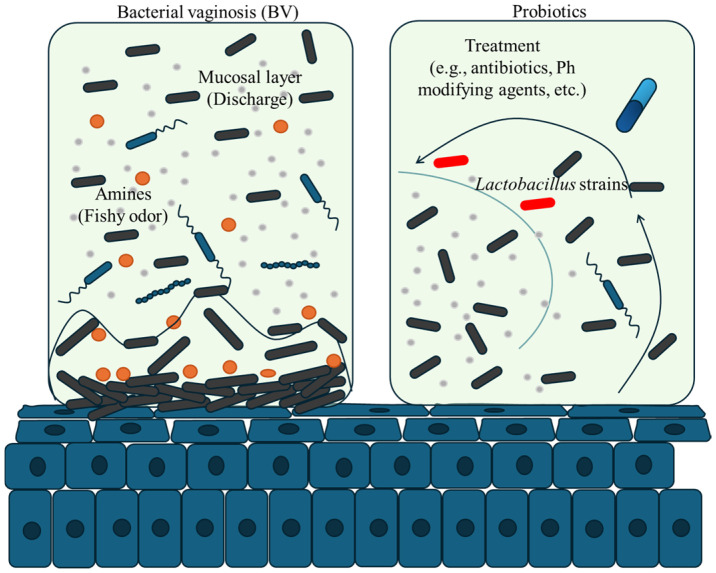
Pathological changes in the vaginal microbiome during BV, where overgrowth of anaerobic bacteria leads to mucosal breakdown, discharge, and a fishy odor. This imbalance reduces Lactobacillus dominance, increasing infection risk. Treatment restores balance by reintroducing Lactobacillus through antibiotics, pH-modifying agents, or probiotics, reducing harmful bacteria and promoting a healthy vaginal environment.

Current treatment options for the BV primarily involve the use of antibiotics such as metronidazole or clindamycin, which target the overgrowth of anaerobic bacteria. These antibiotics can be administered orally or intravaginally, targeting the anaerobic bacteria responsible for the imbalance in the vaginal microbiota. While effective in the short term, these treatments often result in high recurrence rates due to the disruption of the vaginal microbiome. Emerging alternatives include probiotics, which aim to restore *Lactobacillus* dominance; prebiotics to support beneficial bacteria; and novel approaches like phage therapy and biofilm-disruptive agents, but their efficacy has shown mixed results. Additionally, long-term use of antibiotics raises concerns about antimicrobial resistance and potential side effects, such as gastrointestinal disturbances or secondary infections like candidiasis. As such, there is a growing interest in developing alternative and more sustainable therapeutic approaches to address the limitations of current BV treatment regimens; see Figure 1.

### 4.1. Antibiotics

For the initial treatment of BV, antibiotics are usually used for one week and have an efficiency rate between 80% and 90%. However, the cure rate in clinical practice is not higher than 60% after 4 weeks of treatment [7,49]. The recommended antibiotics for BV with a standard course of treatment are outlined in Table 1.

Most females suffering from BV are generally cured after a single treatment employing an antibiotic in a short time frame [11,53,54]. The main complication with current antibiotic treatment is recurrence of BV, accounting for a rate of 50% to 100% even after a treatment for one year [7]. Furthermore, the problem of developing high resistance due to *Gardnerella vaginalis* and *Atopobium vaginae*, which makes antibiotic treatment less sensitive, and the use of them for further treatment of BV. The oral intake of clindamycin and metronidazole disrupts the healthy gut bacteria, while the risk of vulvovaginal candidiasis increases with local use of antibiotics [55,56,57,58]. BODIPY-based supramolecular systems are poised to significantly advance and play a growing role in anti-tumor treatments [47].

### 4.2. Probiotics

The high abundance of vaginal bacteria such as *L. crispatus* has been known to maintain a healthy vaginal condition, whereas high bacterial communities like *L. iners* and non-*Lactobacillus* strains, including the *human papilloma virus* and *Chlamydia trachomatis*, are associated with an increased risk for vaginal infections [31,32,59]. Probiotic treatment has been shown to have long-term benefits, such as a high cure rate and a reduction in recurrence of BV by more than twofold [60,61]. The probiotic treatment regimen for BV is rather safe and shows benefit in both the short and long term. However, many clinical trials and systematic reviews related to probiotic treatment for BV have shown inconclusive results in terms of their efficacy [62]. With probiotics, the major disadvantage is that they have strains of beneficial bacteria but lack other potential benefits, as encountered with bacteriophages or prebiotics, such as stimulating growth as well as colonization of the main beneficial bacteria like *Lactobacillus species*. Apart from resident vaginal bacteria, there are a lot of factors like level of glucose and lactic acid, hormone levels, and, importantly, sexual intercourse, which can influence *Lactobacillus* colonization in the vagina [63,64]. The differences in the structure of genetic and immunological aspects in the human race are another major reason for the lack of success of a single *Lactobacillus* strain as a probiotic fit for all people, due to the large variation in the genome of *L. crispatus* in the vaginal microbiome of different people [65,66].

### 4.3. Prebiotics

Another alternative to treat BV is prebiotics, which consist of compounds that provide nutrients as well as stimulate the flourishing of *lactobacilli* [67]. Studies have already shown the beneficial impact of prebiotics on intestinal health [68,69]. Therefore, prebiotic compounds such as lactitol, lactulose, raffinose, and oligofructose were assessed to determine whether they could stimulate vaginal *lactobacilli* [70]. A summary of prebiotics used in the treatment of BV is presented in Table 2.

### 4.4. Symbiotics

Prebiotics exert their beneficial effects only in the presence of a *Lactobacilli* population; however, in cases of vaginal dysbiosis, the *Lactobacilli* population is often completely depleted, limiting the efficacy of prebiotic treatments. Symbiotics, which combine prebiotics and probiotics, are typically effective in addressing the limitations of prebiotics. This dual strategy leverages the strengths of both components: prebiotics serve as a nutrient source to stimulate the growth and activity of beneficial bacteria, while probiotics introduce live beneficial bacterial strains, such as *Lactobacillus* species, to restore microbial balance. By working synergistically, symbiotics may overcome the limitations of using either component alone, such as the inability of prebiotics to act in the absence of a *Lactobacilli* population or the challenges of probiotics in achieving sustained colonization. This combination has the potential to enhance the stability of the vaginal microbiota, reduce the recurrence rates of BV, and improve overall treatment outcomes [76]. In a study performed by Russo et al., it was demonstrated that the use of probiotics and prebiotics, such as *bovine lactoferrin*, as an adjuvant to metronidazole, showed an improvement in outcomes in a randomized controlled trial with 48 females suffering from recurrent BV [77].

### 4.5. Phage Therapy

Compared to antibiotics, phage therapy in BV offers many benefits, such as self-amplification, high host specificity, high capacity for biofilm degradation, and low toxicity [78,79]. The vaginal *virome* strongly influences the bacterial community structure [80]. During their lifecycle, bacteriophages produce encoded enzymes called *endolysins* and show antibacterial activity by degrading the *tidoglycan* of the target bacterial cell wall [81]. Therefore, these isolated enzymes could be employed as potential drugs to target the primary pathogens that cause BV. Recently, many investigators have been researching a phage-based therapy for the treatment of BV by selectively targeting *Gardnerella* (Vieira-Baptista P). A recently published study by Arroyo-Moreno et al. showed that novel bacteriophage-derived *endolysins* offer viable alternatives to antibiotics in treating BV because they neither cause resistance, like those associated with antibiotics, nor harm beneficial commensal bacteria [82]. Similarly, a recent study performed by Landlinger et al. found that PM-477 eliminates *Gardnerella* from cultures of isolated strains as well as from clinically derived samples of natural polymicrobial biofilms with high selectivity and efficacy and could serve as an alternative to antibiotics in treating patients who frequently experience BV recurrences [83].

### 4.6. Activated Charcoal

Recent research highlights activated charcoal as a viable treatment option for various ailments and injuries [70]. Studies comparing the efficacy of activated charcoal and chloramphenicol in treating BV have demonstrated a reduction in discharge and malodor in both treatment groups. Notably, a 10% activated charcoal solution exhibited maximum efficacy, significantly lowering vaginal pH levels while causing minimal reductions in *Lactobacillus* populations. These findings suggest that activated charcoal may offer an effective and potentially less disruptive alternative to conventional antibiotic treatments for BV [84]. The rationale behind using activated charcoal in the treatment of BV is rooted in its porous nature, which exhibits a lower affinity for binding to *Lactobacillus* species compared to other bacteria. This selective binding property allows it to target pathogenic microorganisms while preserving beneficial bacteria. A clinical trial involving BV patients demonstrated that activated charcoal effectively reduced vaginal pH, a critical factor in restoring a healthy microbiome, while minimally impacting the *Lactobacillus* population, with only a 3.1% reduction observed. These findings highlight its potential as a targeted and less disruptive therapeutic option for BV [6].

### 4.7. Biofilm Disruptive Agents

TOL-463 is basically a vaginal gel considered safe and effective for BV treatment prepared using novel boric acid, having anti-infective properties, and ethylenediaminetetraacetic acid, having antibiofilm activity [85]. A phase II clinical trial conducted on 106 females confirmed that the insert form of TOL-463 showed a 59% cure rate, while the gel form had only 50% at 9–12 days. Further studies have shown that the use of boric acid for the treatment of recurrent BV has an advantage over conventional oral metronidazole treatment [86,87]. The combination of ethylenediaminetetraacetic acid with boric acid has been found to enhance the antimicrobial and also increase antibiofilm potency against *Candida* and *G. vaginalis* without damaging *lactobacilli* [85]. When a proper diagnosis is not feasible, this therapy shows additional benefit by acting not only on biofilms but also on *candidiasis in BV.*

## 5. A Promising Approach Inspired by FMT

The success of FMT as an innovative and safe method for rebalancing the intestinal microbiota has, over time, inspired American scientists to explore its potential application in the treatment of BV [88]. They hypothesized that if stool transplants rebalance the intestinal microbiota, vaginal bacteria transplants could also restore a healthy vaginal microbiota. A study conducted by Delong et al. on 20 females aged 25 to 35 years revealed that a substantial presence of *Lactobacillus crispatus* in the vaginal microbiota significantly increases lactic acid production. This elevated lactic acid content helps maintain an acidic pH, providing a protective barrier against infectious agents and contributing to overall vaginal health [3]. Further, research studies found that the female vaginal wall harbors a large population of *Lactobacilli*, which metabolize glycogen produced by vaginal epithelial cells under the stimulation of estrogen into lactic acid for maintaining an acidic pH of 4.0–4.5 [64,89]. These *Lactobacilli* also form an epithelial mucosal barrier (biomembrane) in the vagina and act as a first-line defense against almost all types of invading pathogens [38]. Yet another key factor for preventing pathogenic organisms, including *Mycoplasma*, *Gardnerella*, *Bacteroides*, and *Streptococcus*, from overgrowing is a low pH caused by lactic acid and the production of H_2_O_2_ and bacteriocins, which are antimicrobial substances [17]. There is greater enrichment of *G. vaginalis*, *Bacteroides*, and *Prevotella* in samples taken from BV patients than in healthy females [90,91]. Several studies also showed that vaginal microbiota yields lactic acid that shows effective anti-inflammatory and antimicrobial activity properties in cervicovaginal epithelial cells and reduces sexually transmitted infections as well as their transmission [38,92,93]. Research in the past has also shown that lactic acid is capable of suppressing both the spread of pathogenic bacteria associated with BV as well as the rate of recurrence of BV [94,95,96]. Interestingly, BV risk in females who lack H_2_O_2_-producing *lactobacilli* was higher after taking antibiotic treatment [5,97,98]. Consequently, several studies have recommended the introduction of exogenous *Lactobacillus* strains as a strategy to restore balance to the vaginal microbiota [99].

Healthy vaginal epithelium also plays a vital role in preventing invasive infections [85,100]. An influential role that is played by the apical layers of vaginal epithelium is to act as an interface between the host and the environment and provide protection against infection in the vagina [101]. The flattened, loosely connected dead cornified cells of the vaginal stratum corneum (SC) lack intracellular organelles, nuclei, DNA, and RNA, leading to non-expression of de novo proteins, which recognize and serve as defense against pathogenic bacteria [102]. This layer also displays distinct features of permeability to cellular and molecular immunological mediators of immune defense and microbes due to a lack of robust intercellular junctions and a complete lipid envelope [103,104]. The loose attachment of the cells provides an environment that promotes endogenous vaginal microbiota while preventing foreign bacteria invasion [100]. The reproductive tract in females has a mucosal immune system that is uniquely adapted to manage commensal bacteria, sexually transmitted pathogens, allogeneic spermatozoa, and the immunity of the fetus [105]. A previous study has shown that pivotal cells of the innate and adaptive immune systems in the female reproductive tract are antigen-presenting cells and functionally respond to various antigens in the fallopian tubes, uterus, and cervix. These cells provide protection to neutrophils, macrophages, natural killer cells, and epithelial cells through Toll-like receptors by producing chemokines and cytokines that deploy and also activate immunocytes, virucides, and bactericides that provide protection, especially when sex hormones downregulate adaptive immunity [106,107].

## 6. VMT as an Emerging Concept for BV

Bacteria in the gut and vaginal cavity play an essential role in maintaining physiological and nutritional homeostasis, which is vital to human health. The landscape of gut bacteria in the human gut, including bacteria, archaea, and eukaryotic microorganisms, and *Lactobacilli* in the vagina, is largely determined by the host genotype, the colonizing history, the host bionomy, and other environmental factors [108,109]. There are a variety of key roles that gut bacteria play, such as digestion, metabolizing drugs, facilitating immunity, competing with pathogens by occupying niches, and promoting intestinal angiogenesis [110,111,112,113]. The vaginal cavity also hosts a highly diverse microbiota population that maintains a balanced ecological system by promoting good health through physiologic, metabolic, and immune homeostasis [114,115]. Vaginal flora exhibits cooperative and competitive interactions with one another, as well as symbiotic relationships with the host tissue and organ. However, not all microbial species are beneficial, and the enrichment of some specific bacteria can be problematic [116,117]. A previous study suggested limiting the use of VMT to females who were negative for *G. vaginalis* to avoid risk for BV [118]. Generally, females at any stage of their lives may suffer from vaginal infections caused by disrupted gut bacteria [119,120,121]. Surprisingly, the FMT process has shown encouraging results for some diseases resulting from disturbance of whole microbial communities as compared to single or combination forms of probiotics [122]. Although many probiotics have also been used for BV, most of them have shown auxiliary effects with antibiotics but have failed when used alone. Therefore, VMT could be a very effective treatment for BV. This potential stems from the physiological and pathogenic similarities observed in conditions caused by the overgrowth of pathogenic microorganisms in both the intestinal tract and the vaginal cavity.

Despite the promising outcomes reported in the initial proof-of-concept study by Lev-Sagie et al., it is important to emphasize that the current clinical evidence supporting VMT remains extremely limited. To date, published human data are derived primarily from small pilot studies involving a very small number of participants, and therefore cannot yet be generalized to broader patient populations.

### 6.1. Preconditions Required for VMT

Preconditions for VMT are important to its effectiveness and safety. To ensure the recipient’s safety, the donor must first be thoroughly screened for transmissible illnesses, such as STIs, BV, and other microbial imbalances. Donors should also be assessed for overall vaginal health, with a focus on the presence of beneficial bacterial strains, particularly *Lactobacillus* species, which are critical in maintaining vaginal microbiota balance. Recipients should have a thorough clinical evaluation to establish eligibility and discover factors causing their microbial dysbiosis. Pre-treatment regimens, such as antibiotics or antifungals, may also be required to eliminate pathogenic organisms and prepare the vaginal environment for transplantation. In short, patient screening, inclusion criteria, exclusion criteria, and the consent process for participants for VMT must be met before joining the trial.

#### 6.1.1. Diagnosis of BV

In order to diagnose BV as per Amsel criteria, a minimum of three of these four symptoms or signs are required: homogeneous, thin, white discharge, a vaginal fluid having pH > 4.5, a fishy odor in vaginal discharge before or after adding 10% potassium hydroxide (the whiff test); and on microscopic examination, >20% of the vaginal epithelium has adherent *coccobacilli* (clue cells) [123]. According to Hay-Ison criteria, if the microbiome of an individual is *Lactobacillus*-predominant, it would be classified as normal, while *coccid-bacillary*-dominated or intermediate would be considered positive for BV [9].

#### 6.1.2. Inclusion Criteria for Recipients

The inclusion criteria for study candidates are recurrent BV, with at least four times within the current year, in individuals aged 18–50 years. Further, the individual depends upon antibiotic therapy twice weekly to remain symptom-free, or if they have a documented history of prior BV recurrence, and are now again showing the recurrence in a period of 2 months or less, even after following antibiotic treatment [9].

#### 6.1.3. Exclusion Criteria for Recipients

The key criteria used for study candidates include non-pregnant females, free from planned pregnancy in the coming year, free from infection, including HIV, hepatitis B, hepatitis C, or syphilis. Study candidates are also free of cervicovaginal infections, including *Chlamydia trachomatis*, *Neisseria gonorrhea*, *Trichomonas vaginalis*, and *Mycoplasma genitalium*, *confirmed* by PCR testing. The study candidates receive standard recommended treatment after confirming a positive diagnosis for these diseases. Study recipient candidates free from *human papillomavirus* and a cervical cytology screening test (Pap test) confirmed by PCR-based screening. The vaginal cultures of the recipient are free from yeast, bacteria (*streptococci* groups A, B, C, and G), and other tests, including urinalysis, urine cultures, and serology analysis for hepatitis A, B, and C, HIV, *Treponema pallidum*, *cytomegalovirus* (CMV), and *herpesviruses* [9].

#### 6.1.4. Inclusion Criteria for Donors

Inclusion criteria used for donor selection include age between 18 and 50 years, being premenopausal, and having a negative history of vaginal symptoms. Furthermore, they should not be positive for BV and other forms of vaginitis, verified through their history, gynecological examination, PCR test, microscopic examination, and culture of vaginal secretion. Donors must be free of potentially serious infections, including group B *Streptococcus* (GBS) or *Streptococcus agalactiae* and CMV, and they must not have engaged in sexual intercourse in the week preceding of vaginal fluid collection [9]. The key instruction of the VMT protocol is that recipients must not engage in sexual activity for one month. Further, the recipient should refrain from bathing for at least seven days. Moreover, the recipient should also avoid douching, intravaginal medication, systemic antibiotics for one month, and probiotics for one year following VMT.

#### 6.1.5. Exclusion Criteria for Donors

Donor candidates are excluded from the study if they are identified as BV positive or even infected with BV in the last 5 years or if they have a history of recurrent BV and cervicovaginal sexually transmitted infections, such as *M. genitalium*, *C. trachomatis*, *T. vaginalis*, and *N. gonorrhea.* Furthermore, study participants are also excluded if an individual has a history of recurrent candida vulvovaginitis and urinary tract infections IV, or syphilis, and shows the presence of *streptococci* groups A, C, or G and a positive HPV test. Moreover, if the donor used any antibiotics or systemic medication in the month preceding vaginal fluid collection, used herbal or homeopathic remedies, or used probiotics (orally or vaginally) are also excluded from the study. Individuals with a history of disease like anogenital dysplasia, anogenital HPV, anogenital herpes, vulvar or vaginal disease, cancer, abnormal urinalysis, infection, pregnancy, seropositivity to hepatitis C, hepatitis B, HIV, syphilis, and having long-term treatment medical and sexual history with a clinician [9].

### 6.2. VMT Procedure

The most appropriate time for collecting vaginal secretions from donors is day seven of the menstrual cycle. The samples should be taken from the upper vaginal and cervical fornices and must avoid contact with the cervix region. Importantly, the broad end of a flat Ayre’s spatula should be inserted for the collection of vaginal secretion. This spatula has a vaginal shape and offers the advantage that it does not absorb vaginal secretion and does not cause injury to the vagina. This technique is also used to collect samples for molecular analysis using the ESwab Multiple Specimen Collection and Transport System (COPAN) and to store samples at −80 °C collected as part of VMT sampling. After sampling, the collected vaginal discharge must be evaluated for pH and microscopic examination. The vaginal fluid is transferred to the posterior fornix of the recipient’s vagina after diluting with 1 mL of sterile saline without using a speculum within 60 min of collection. The transplant procedure can be applied to the recipient at any phase of the menstrual cycle, excluding the menstrual period [9].

### 6.3. Post-VMT Follow-Up

At each examination, patients must undergo a gynecological examination and a microscopic examination of vaginal secretion. If cytology or HPV tests show abnormal results, a colposcopy must be performed before VMT, as recommended by the American Society for Colposcopy and Cervical Pathology (ASCCP) guidelines [124]. Those patients with normal cytology who have a negative HPV test must also be screened again by cytology after one year, as recommended by ASCCP. Generally, the chances of infection become high after VMT if the recipient maintains sexual relations with their partner. Therefore, these infections could not be attributed to VMT, and there is no need for routine tests [9].

### 6.4. High-Throughput 16S rRNA Gene Amplicon Sequencing

Vaginal microbiome samples from donors and recipients must be sequenced with 16S ribosomal DNA (rDNA) sequencing to identify the changes at the genus level. DNA extraction should follow a standard protocol, followed by 500 bp paired-end sequencing (Illumina MiSeq). Amplicons spanning variable region 4 (V4) of the 16S rDNA gene should be generated using the following barcoded primers: Fwd 515F, AATGATACGGCGACCACCGAGATCTACACTATGGTAATTGTGTGCCAGCMGCCGCGGTAA; Rev 806R, CAAGCAGAAGACGGCATA. The shotgun metagenomic sequencing technique is used for the evaluation of samples collected from all donors and recipients to see the changes in the vaginal microbiome of BV patients at the species level [9].

### 6.5. Microbial Bioinformatics Analysis

The analysis of sequencing data requires robust and modern bioinformatics tools to ensure accurate and reproducible results. Using appropriate software such as QIIME (v2) or DDA2, enable high-resolution identification of microbial communities by generating Amplicon Sequence Variants (ASVs). QC and Trimmomatic should be used to trim and align paired ends, followed by clustering into OTUs (Operational Taxonomic Units) with 97% similarity. The rarefaction method should be used to exclude samples with insufficient read counts. Alpha diversity and beta diversity estimators must be calculated. Principal-coordinate analysis with UniFrac distances can be used to distinguish the microbiomes of the BV patients from healthy individuals on the basis of different clustering patterns of bacteria. The shift in the microbiota composition of recipients before and after VMT can be scored by assessing Bray–Curtis (BC) dissimilarity and then correlated with Amsel’s criteria. Post-VMT, the vaginal microbiota is expected to exhibit a substantial increase in the abundance of *Lactobacillus* species—notably *Lactobacillus crispatus* and *Lactobacillus jensenii*—coupled with a significant depletion of BV-associated taxa, such as *Gardnerella*, *Prevotella*, *Fannyhessea*, and other anaerobic genera. Clustering patterns in PCoA plots should demonstrate distinct separation between BV-associated and healthy microbiota, with the latter showing no association with Amsel-diagnosed features. Statistical significance of microbiota shifts can be determined using permutational analysis of variance (PERMANOVA) based on Bray–Curtis dissimilarity (*p* < 0.05). In addition to taxonomic profiling, functional analyses of the microbiome provide deeper insights into the effects of VMT. Pathway analysis using the Kyoto Encyclopedia of Genes and Genomes (KEGG) can identify critical functional differences between BV-associated and healthy microbiomes. Pathways related to lactic acid metabolism, quorum sensing, microbial pathogenesis, and epithelial barrier integrity are of particular interest. Functional profiling typically reveals distinct clusters, reflecting the restoration of a healthy vaginal microbiome post-VMT [9].

## 7. Readdressing Challenges in VMT

The therapeutic success of FMT for CDI prompted researchers to examine its feasibility for more prevalent antibiotic-resistant bacterial infections, like VMT. This process involves the transfer of the complete community of the vaginal microbiome from a healthy donor into the patient to remodel the microbial diversity of beneficial bacteria. Research of this kind is complex and presents many unforeseen challenges. First, longer phase studies involving a larger cohort of donors and patients, as used in FMT, have also been required in VMT to ascertain the safety of this technique before confirming its efficacy [125].

One study led by Lev-Sagie and colleagues reported that four out of five individuals with recurrent BV who received microbiome transplantation showed full remission from BV. More importantly, no pharmacologic interventions were required in patients following transplantation, and no side effects were experienced by any patients. In the initial phase, identifying donors who meet all standard criteria may be the biggest challenge. Owing to the unique physiology of the vagina in humans in comparison to other animals, it is not possible to conduct in vivo investigations in the early phases of BV. Since vaginal fluid needs to be administered directly to the patient, a very thorough donor screening is a prerequisite for this technique. Another issue involves the timeline, as in this process, investigators collect donor vaginal fluid for a month, during which donors must abstain from sexual activity. Further, a randomized, placebo-controlled trial must be conducted for this research work, and the enrollment of potential VMT recipients is a very difficult and complicated task. Moreover, a patient with a recurrent BV history with three or more episodes on record within the previous 12 months is a stringent inclusion criterion. However, females who have a long-standing history of recurrent BV are unlikely to participate in the randomized trial of the placebo group. Even during the VMT procedure, some patients may show incidents of immunological rejection or infection. Before moving on to VMT, this technique requires genomic sequencing of the donor as well as the recipient, which requires significant effort. To perform VMT, both the donor and recipient need genomic sequencing, which requires substantial effort. Further, after VMT, a routine physical examination must be performed to check the therapeutic effect on BV and also to ascertain the health status of the individual, and, finally, to know whether the VMT in the patient is working effectively or not. In addition, regular review of the microbiota of the vagina in the recipient, along with their clinical index, is also necessary to ensure that the reinstatement of beneficial vaginal microbiota is accomplished. This regular monitoring is very complex and is required for each individual due to having a different immune response and unique microbiota in the vagina. There are ethical issues with VMT, since people are generally reluctant to accept other people’s vaginal microbiota. The main challenge of VMT is the social stigma regarding their acceptance in society.

In addition to ethical concerns and issues of social acceptance, several biological uncertainties present significant challenges for the clinical translation of VMT. One major concern is the potential long-term risk of transmitting unidentified or currently uncharacterized pathogens, including viruses, bacteriophages, or antimicrobial resistance genes, despite rigorous donor screening. Unlike conventional antimicrobial therapies, microbiota transplantation introduces a complex and dynamic microbial ecosystem whose full functional repertoire may not be completely understood at the time of transfer.

Another critical challenge relates to colonization resistance and the long-term stability of donor-derived microbiota within the recipient’s vaginal environment. Host-specific factors such as immune responses, hormonal fluctuations, epithelial barrier integrity, sexual activity, and pre-existing microbial communities may influence whether transplanted microbiota can successfully engraft and persist. Failure of durable engraftment could lead to transient benefits followed by relapse or unpredictable shifts in microbial composition. These uncertainties highlight the need for long-term surveillance, standardized monitoring protocols, and cautious interpretation of early clinical outcomes associated with VMT. However, the engraftment of microbiota of healthy individuals to patients has broad potential outcomes. The situation may be such that even after VMT, the recipient, compared to healthy individuals, shows reduced bacterial diversity. A major stumbling block in VMT is its high degree of uncertainty. Hence, regular inspections are essential to check whether the bacterial diversity is becoming low or an overabundance of unfavorable bacteria, which may cause harm to recipients. The summary of clinical results of recent microbiome transplantation is provided in Table 3.

The above discussion, after underpinning current evidence, concludes that although VMT does not seem to be an obvious standalone remedy for BV. A single study suggests a cure rate of 75% for recurrent BV following VMT. Otherwise, many studies on BV are currently in the pipeline as clinical trials and will take time to complete. Like the FMT database, human VIRGO (vaginal non-redundant gene catalog) is now available as a central reference database for the characterization of the vaginal microbial gene content of individual bacterial species situated in the vagina. Moreover, these alternative treatments still require further research to establish their efficacy. Hence, it is necessary to accelerate the progress of existing clinical trials and conduct more studies on VMT to make sure the treatment is safe and effective.

## 8. Conclusions

A significant decline in the cost of microbiome analysis, together with major technological advances, has accelerated microbiome research in recent years and improved understanding of the role of host genetics, immune responses, and environmental factors in BV. Consequently, multiple therapeutic approaches, including antibiotics, probiotics, prebiotics, symbiotics, and novel antimicrobial strategies, are currently being explored for BV management. However, probiotic-based interventions, whether used alone or in combination with antibiotics, remain limited by inconsistent long-term efficacy and difficulties in achieving stable Lactobacillus colonization in the dysbiotic vaginal environment.

These limitations have driven interest toward more comprehensive approaches, such as VMT, inspired by the clinical success of fecal microbiota transplantation in recurrent Clostridioides difficile infection. Despite its promise, VMT remains firmly in the experimental stage, with current evidence restricted to small pilot studies and ongoing clinical trials. As such, its effectiveness, durability, and safety cannot yet be generalized, and VMT is not ready for routine clinical application.

Future research should prioritize well-designed, large-scale randomized controlled trials with extended follow-up to establish robust evidence for VMT. Key research directions include defining the optimal characteristics of donor microbiota, standardizing transplant preparation and delivery methods, and implementing long-term monitoring systems to assess engraftment stability, recurrence rates, and potential delayed adverse effects. Furthermore, advances in microbiome profiling and bioinformatics may enable personalized VMT strategies, such as donor–recipient matching based on the recipient’s baseline microbial composition and host-specific factors. Collectively, sustained international collaboration and coordinated clinical research efforts will be essential to determine whether VMT can evolve from an experimental intervention into a safe, effective, and personalized therapeutic option for recurrent BV.

## Figures and Tables

**Table 1:** Treatment options for BV (non-pregnant females or persons with a vagina).

S. No	Therapies	Dose	Duration	Adverse Effects	References
1.	Metronidazole	400 mg orally every 12 h or 0.75% gel 5 g once daily	5–7 days 5 days	Metallic taste, nausea, and transient neutropenia	[46]
2.	Clindamycin	300 mg orally every 12 h or 100 mg intravaginally once daily	7 days 3 days	Vulvovaginal candidiasis and gastrointestinal side effects.	[50]
3.	Tinidazole	2 g orally once daily 1 g orally once daily	2 days 5 days	Metallic/bitter taste, nausea, and weakness or fatigue	[50]
4.	Secnidazole	2 g packet (orally) once		Vulvovaginal candidiasis. Headache, nausea, diarrhea, and abdominal pain	[51]
5.	Dequalinium chloride	10 mg tablet intravaginally	6 nights	Product use is limited	[52]

**Table 2:** Effect of prebiotic treatment on BV.

S. No.	Prebiotics	Beneficial Effects	Adverse Effect	References
1.	Oligosaccharides	Selectively help in promoting the enrichment of *lactobacilli* Increase lactic acid production Prevent the growth of anaerobic bacteria by inhibiting adhesion and replication through the secretion of antibacterial substances.	Not observed	[71]
2.	Vaginal sucrose gel	The therapeutic cure rate was 61% after 21–35 days of treatment At 5–7 days, the Nugent score showed significantly higher levels of *lactobacilli* in the sucrose gel group treatment than in the metronidazole group.	Promote the development of candidiasis	[72]
3.	Disaccharide lactulose	Promote the enrichment of vaginal *lactobacilli,* including *L. crispatus*, and non-stimulating *C. albicans* and other harmful bacteria spotted in BV.	Not observed	[73]
4	Trifolium pratense (red clover) extract and Galacto-oligosaccharides	Nugent score ≤ 3	Not observed	[74]
5.	*Lactoferrin*	Improve the BV by gradually increasing of *lactobacilli*	Not observed	[75]

**Table 3:** Summary of clinical results of recent microbiome transplantation studies.

Citations ClinicalTrials.gov Identifier	Population	Study Focus	Primary Cure Rate
[126]	*N* = 46	FMT vs. Antibiotic	91%
[127]	*N* = 180	FMT Delivery Method	82%
[128]	*N* = 72	FMT Material Processing	100%
[100]	*N* = 15	Topical Microbiome Transplant	75%
[9]	*N* = 5	VMT for Recurrent Bacterial Vaginosis	75%
NCT04046900	*N* = 134	VMT for Recurrent Bacterial Vaginosis	Primary completion in December 2024
NCT04517487	*N* = 100	Biological: Vaginal Microbiome Transplantation	Primary completion in December 2024

## Data Availability

No new data were created or analyzed in this study. Data sharing is not applicable to this article.
